# Effect of early photostimulation at 15-weeks of age and everyday spin feeding on broiler breeder performance

**DOI:** 10.1016/j.psj.2022.101872

**Published:** 2022-03-24

**Authors:** A.P. Benson, R.H. Blocher, Z.R. Jarrell, C.K. Meeks, M.B. Habersang, J.L. Wilson, A.J. Davis

**Affiliations:** University of Georgia, Athens, GA 30602, USA

**Keywords:** feed restriction, management, welfare, egg production, fertility

## Abstract

To prevent broiler breeders from growing too quickly and becoming too large for optimum reproduction, their dietary intake is restricted. While current restricted feeding programs, such as skip-a-day feeding (**SAD**), improve the economic efficiency of broiler breeder operations, this management practice impacts bird welfare. There is an interest in finding strategies that could reduce the impact of feed restriction during broiler breeder rearing. This research investigated the effects of feeding pullets on an advanced growth curve for early photostimulation at 15 wk (**15P**) or standard growth curve for photostimulation at 21 wk (**21P**), using either an every-day-spin feeding program (**EDS**) or SAD feeding, on the reproductive parameters of broiler breeder hens in a 2 × 2 factorial arrangement. Overall, advancing the growth curve (15P) decreased blood corticosterone levels compared to 21P, but EDS resulted in higher blood corticosterone levels compared to SAD. At the end of rearing in both 15P and 21P, EDS pullets weighed less than SAD pullets. The onset of egg production was 20 and 24 wk of age for the 15P and 21P hens, respectively. Despite an earlier onset, 15P hens did not produce more eggs than 21P hens through 65 wk of age. Egg weight was reduced for 15P compared to 21P until 30 wk of age. The 15P hens had a greater number of double yolk eggs than the 21P hens. Fertility and hatch were not impacted by the advanced growth curve and early photostimulation. Although the current research indicates the potential to reduce feed restriction associated welfare issues by rearing broiler breeder pullets for an earlier photostimulation onset, further research in needed to determine if this management technique can be improved to optimize hen reproductive efficiency.

## INTRODUCTION

Broiler breeder pullets are fed to meet recommended growth curves using feed management strategies intended to prevent excessive growth while providing the nutrients required for proper skeletal development and body condition at target BW (2.1 kg) by the age of photostiumlation at 20 to 22 wk of age. Although the genetic potential of broiler growth has continually improved over the last 50 yr (Zuidhof et al., 2014), the recommended BW profiles of broiler breeders has not been changed (Renema et al., 2007a), so the resulting gap between breeder BW and broiler growth potential requires strict feed restriction for broiler breeders. To achieve a proper growth curve during rearing, feed quantities for broiler breeders are reduced, and this leads to increased competition for feed, resulting in low BW uniformity.

To prevent poor BW uniformity, restriction can be implemented in a quantitative or qualitative way. Quantitative restriction methods consist of limited amounts of standard diets usually offered to the pullets in a single meal while qualitative restriction involves the use of feeds diluted with non-nutritive fillers or low energy and protein feed ingredients to increase volume and allow larger amounts and/or multiple meals to be fed which can improve flock uniformity and results in comparable egg production to qualitative feed restriction programs (Tolkamp et al., 2005). Both quantitative and qualitative feed restriction methods are associated with poor bird welfare outcomes (Sandilands et al., 2005; Tahamtani et al., 2020). Feed restricted broiler breeders show behavioral idiosyncrasies that are indicative of hunger and frustration of the feeding motivation, like hyperactivity, stereotyped object pecking, and overdrinking in concert with elevated plasma corticosterone (Savory et al., 1993; Hocking et al., 1996; de Jong et al., 2002, 2005; Mench, 2002; Mormede et al., 2007). Despite exhibiting behavior stress, feed restricted broilers have decreased mortality (Bruggeman et al., 2005) and produce more eggs (McDaniel et al., 1981; Yu et al., 1992; Heck et al., 2004; Bruggeman et al., 2005; Onagbesan et al., 2006) then full-fed broiler breeder hens.

The most widely used quantitative method in the United States is some form of skip-a-d (**SAD)** feeding where pullets are provided with 2 d worth of feed every other d. Although quantitative restriction prevents the major negatives effects of ad libitum feeding, it alters the normal physiology of the birds resulting in abnormal behaviors and changes in plasma corticosterone levels (Savory and Lariviere, 2000; Sandilands et al., 2005, 2006; Morrissey et al., 2014). For feed restriction programs such as SAD feeding, feed distribution is typically automated by means of mechanical trough feeders that rapidly distribute the feed to the flock so all birds are given a chance to access feeder space which will improve BW uniformity. More recently, broadcasting feed or everyday spin feeding (**EDS**) on the litter has been adopted as a feed distribution method in several countries to mimic natural food foraging behavior, and this feeding technique has resulted in less mortality, more uniform BW, reduction in behavioral idiosyncrasies associated with hunger frustration and no loss in subsequent egg production (de Jong, et al. 2005; EFSA, 2010; Zuidhof et al. 2015).

Another possible route to reducing stress associated with feed restriction during rearing is to increase the amount of feed allocated on an advanced growth curve to reach target BW for dissipation of juvenile photorefractoriness (**PR**) and early photostimulation (**PS**). The amount of time needed to dissipate juvenile PR in broiler breeders is modified by the degree to which growth is restricted, with a relaxation of feed restriction allowing the attainment of sexual maturation at a younger age (Lewis et al., 2007). Therefore, an accelerating growth curve advances the dissipation of juvenile PR, and Lewis et al. (2007) produced a model that indicated the lower limit for uniformly stimulating sexual development within a flock that has been allowed maximum or near maximum growth is around 10 wk of age. This timeframe agrees with the 2 mo needed to dissipate juvenile PR in ad libitum fed seasonal birds (Follett and Pearce-Kelly, 1991). Several studies have demonstrated that broiler breeders photostimulated at 15, 17, or 18 wk of age results in earlier sexual maturation (Ciacciariello and Gous, 2005a; Renema et al, 2007b; Pishnamazi et al., 2014; van der Klein et al., 2018). Advancing the growth curve advances the dissipation of the photorefractory state, so obtainment of target BW (2.1 kg) can result in earlier photosensitivity (Lewis et al., 2007; van der Klein et al., 2018). Advancing the growth curve for obtainment of target BW at 15 wk of age instead of the typical 21 wk of age would likely reduce stress as the degree of feed restriction would be significantly less.

Therefore, the basis of this research is a 2 × 2 factorial designed experiment with 2 rearing growth curves/PS ages and 2 different feeding regimes. This research looked at the impact of 1) advancing the age of PS from 21 to 15 wk of age with the corresponding attainment of the target body weight (2.1 kg) on reproductive efficiency in broiler breeders, 2) the effectiveness of the more natural feeding practice of EDS feeding rather than SAD feeding on birds under these 2 different growth curves. It was hypothesized that feeding pullets on an advanced growth curve for obtainment of target BW and PS at 15 wk of age (**15P**) will result in improved BW uniformity and blood corticosterone levels during rearing, earlier onset of sexual maturation and expansion of the egg production period when compared to birds grown on a conventional growth curve to obtain target BW for PS at 21 wk of age (**21P**). In addition, it is hypothesized that EDS feeding during rearing will improve flock uniformity and positively impact bird welfare, when compared to SAD feeding.

## MATERIALS AND METHODS

### Rearing Management of Birds

A total of 2,400 female one-d old Cobb 500 broiler breeder chicks were reared in an. environmentally controlled solid side wall poultry house at the University of Georgia Poultry Research Center that contained 6 rooms with each room containing 2 pens. Twelve pens (7.32 × 4.6 m^2^, 200 pullets per pen) with fresh pine shaving litter were allocated into one of 4 groups (3 replicate pens and 600 pullets per treatment): EDS - 15P, EDS - 21P, SAD - 15P and SAD - 21P. Rearing treatments were separated into different rooms to eliminate variation incurred through SAD birds either hearing or seeing the EDS birds eat on the SAD off-feed d. Birds were fed a standard corn/soybean meal starter mash diet (2,910 kcal/kg, 18% CP) ad libitum through 2 wk of age, then they were switched to a standard pelleted breeder developer diet (2,835 kcal/kg, 14.5% CP) on either a SAD schedule using chain-feeders, or an EDS schedule with hand broadcast feeding for improved distribution. The total amount of feed fed during each 48-h period for the SAD and EDS treatments was equal. Pullets were fed to reach a target BW of approximately 2,100 g, at either 15 or 21 wk of age. Both the average allocated daily feed amounts and weekly target body weights for each growth curve (15 wk and 21 wk) during rearing is presented in Table 1. Water was provided ad libitum from nipple drinkers. Day length was decreased linearly from 24 h light to 8 h light:16 h dark from 2 to 7 d of age and remained there until PS at 15 or 21 wk of age. The temperature of the rooms was maintained at 32.2°C for the first wk, and then the temperature was decreased by approximately 2.8°C every wk thereafter until the target temperature of 21°C was reached. Pullets were wing-banded at 3 wk of age and weighed every 3 wk, to adjust feed allotments to maintain target growth curves.

**Table 1:** Average allocated daily feed amounts and target growth curve for each week during rearing to obtain target body weight for photostimulation (PS) at 15 wk (15P) or 21 wk (21P).

	15P	21P	15P	21P
Wk of age	g/bird/d		Target growth curve (g)	
3	49	36	400	375
4	53	39	540	400
5	56	40	690	500
6	57	41	820	580
7	62	42	950	675
8	65	44	1,100	775
9	70	46	1,240	875
10	84	51	1,390	975
11	89	54	1,540	1,075
12	94	57	1,680	1,175
13	98	60	1,830	1,275
14	102	63	1,980	1,375
15	102	66	2,100	1,475
16		70		1,600
17		75		1,720
18		80		1,855
19		90		1,995
20		100		2,100

A total of 360 male one-day old Cobb 500 broiler breeder chicks were divided and reared in 4 floor pens each measuring 3.66 × 4.55 m^2^. Cockerel rearing treatments paralleled the pullet rearing groups and they were fed the same diets. Cockerel rearing management was exactly as previously described for the pullets, except that the 4 floor pens were all in the same room and thus the SAD birds could visualize EDS feeding on the skipped d.

### Breeding Management of Birds

At 14 wk of age all the pullets from the EDS - 15P and SAD - 15P treatments were individually weighed. Based on these weights, and the rearing feed distribution treatment, pullets were selected at 15 wk of age to create 9 pens that had an equal weight profile for each of the 2 treatments. Thus, a total of 18 breeder pens were created, and each pen had 38 pullets and 4 roosters. The 18 pens were distributed into 3 separate rooms with each room having 3 EDS - 15P and 3 SAD - 15P treatment pens. This same process was repeated at 20 wk of age for the pullets from the EDS - 21P and SAD - 21P treatments.

Each breeding pen measured 3.65 m by 2.75 m, and the floor space of each pen consisted of 2/3 elevated slates and 1/3 pine shavings litter. The slat section contained 10 nipple drinkers, a 6-hole nesting box, and 4 hen feeders with rooster exclusion grills. A rooster feeder was hung over the pine shavings and kept elevated enough during feeding time to restrict access to hens. During the production period, regardless of rearing treatment, all birds were fed every d. Feed was allocated when the lights came on at 6:30 am every morning, and feed type was switched from developer pellets to layer pellets (2,865 kcal/kg, 14% CP) at wk 18 and 24 of age for the 15P and 21P birds, respectively. Photostimulation d length was 14 h light:10 h dark. Ratio of males to females was held constant at 10% by replacing male mortality with extra males that had been reared for the 15P and 21P treatments. In order to adjust feed allotments to maintain egg production and the breeder recommended BW growth curve, 3 pens from each rearing treatment were weighed weekly from the time of PS until the conclusion of the experiment at 65 wk of age. The 3 pens selected per treatment for weighing each wk rotated such that all 9 pens from the individual rearing treatments were weighed every 3 wk. All hens were weighed on wk 20, 30, 41, 50, 55, 60, and 65 and wk 26, 30, 43, 51, and 61 for the 15P and 21P treatments, respectively.

Eggs were collected 2 times/d and egg weights were recorded for 12 of 36 pens daily (3 from each rearing treatment). The eggs from all 36 pens were weighed once every 2 wk. Hen-housed egg production **(HHEP)** was calculated weekly using daily egg counts. The number of normal (hatching eggs), floor, abnormally shaped, cracked, double-yolked, dirty, and total eggs were recorded from each pen. A maximum of 90 hatching eggs was collected from each pen every 3 wk and incubated in Natureform incubators (Natureform Hatchery Systems, Jacksonville, FL). Eggs were collected and stored between 18.3 and 19.9°C for up to 7 d before each incubation period. Eggs were candled on d 12 of incubation, transferred for hatching on d 19 and hatched on d 21 of incubation. Temperature settings for incubation were 37.8^°^C from 0 to 18 d and 37.2^°^C from 19 to 21 d. Relative humidity settings were 53% from 0 to 19 d and 70% from d 20 to 21. During candling, transfer and after hatching, eggs were characterized as being infertile, cracked, contaminated, or containing early and mid-dead embryos (0–12 d) or late-dead embryos (13–21 d). Eggs that were cracked in transfer were removed from data set. After hatching, the number of live and dead-in-shell and live and dead hatched chicks were determined.

All animal procedures were approved by the Animal Care and Use Committee of the University of Georgia.

### Corticosterone ELISA

At 10, 15, 20, 24, and 26 wk of age, 30 birds from each treatment were randomly selected for blood sample collection. Blood samples were collected at 7 h (EDS and SAD birds), 24 h (EDS only), and 48 h (SAD only) post-feeding during the rearing period, and at 7 h post-feeding in the production/breeding period. The 7 h post-feeding time was selected to compare the pullets when the excitement of feeding had past and while the birds would still have food in their digestive tract. The 24 and 48 h post-feeding times were selected as these times would represent the longest metabolic fasting stress for the EDS and SAD pullets, respectively. Approximately 3 mL of blood was collected from the brachial vein and immediately placed into individual glass vacutainers (Becton, Dickinson, and Co., Franklin Lakes, NJ) containing EDTA as an anticoagulant and stored on ice. Blood samples were collected within one minute of physical contact with each hen to avoid corticosterone levels being influenced by handling stress (Romero and Reed, 2005). Blood samples were centrifuged at 1,000 × *g* at 4° C for 10 m. Plasma was collected from each sample and frozen at −80° C.

Following ether extraction (Pusch et al., 2018) of steroid hormones, the corticosterone levels in plasma were determined using an ELISA assay (Cayman Chemical, Ann Arbor, MI) according to the manufacturer's instructions. A 50 µL volume was used for each duplicated sample. Each plate was read at a wavelength of 405 nm in a SpectraMax ELISA plate reader (San Jose, CA). A total of 7 plates were used to accommodate all the samples and the inter- and intra-assay % CV were below 15 and 10%, respectively.

### Statistics

One-way ANOVA was used to detect significant weekly or overall experimental period differences among and between the different treatments. Data was first tested for an interaction (SAD vs. EDS×15P vs. 21P) using effects test in IBM SPSS Statistics for Macintosh (Version 27.0, Armonk, NY). When no interaction between the 2 factors was found, the data were subjected to one-way ANOVA and analyzed via Tukey-Kramer HSD means comparison (Driscoll, 1996). Treatment was source of variation, and a statistic was considered significant if it was below *P* = 0.05.

## RESULTS AND DISCUSSION

### Rearing: BW, Uniformity, and Blood Corticosterone

#### 15P vs. 21P Rearing

The welfare of broiler breeders is impaired due to hunger associated with severe feed restriction during rearing (Savory et al., 1993; Hocking et al., 1996; Mench, 2002; Aranibar et al., 2020). There is an established relationship between the level of feed restriction and hunger with plasma corticosterone concentration (Hocking et al., 1996; Kurbikova et al., 2001; de Jong et al., 2003). In the current research, both rearing feed strategy (**RS**), either SAD or EDS, and growth curve for PS, at either 15 or 20 wk, had an impact on blood corticosterone (Table 2). The 15P growth curve had significantly lower plasma corticosterone levels, when compared to 21P pullets, at both 10 wk and 15 wk, except 7 h post-feed on wk 15 (Table 2). This result confirms the research hypothesis and demonstrates that increasing the feed allotment with the 15P growth curve reduces the severity of hunger associated stress during rearing and agrees with de Jong et al. (2003) who reported a higher level hunger associated stress with higher levels of feed restriction. This increased daily feed allotment to maintain the advanced growth curve for 15P pullets also resulted in improved BW uniformity relative to the 21P pullets, between 8 and 15 wk of age (Table 3), when feed intake in the 21P growth curve is the most restrictive (de Jong and Jones, 2006). This increased feed allotment allowed for increased feed cleanup time, and thus allowing more timid pullets access to feed. Overall, the 15P pullets, when compared to the 21P pullets, had improved welfare, as assessed by blood corticosterone levels, and improved BW uniformity during the shortened rearing phase. The results indicate that the severity of feed restriction, to reach target BW for PS at 15 wk of age can be relaxed enough to obtain improved BW uniformity and welfare during rearing.

**Table 2:** Plasma corticosterone concentration in broiler breeder pullets raised using different rearing strategies (RS), everyday spin feeding (EDS) or skip-a-day feeding (SAD), to reach target body weight for photostimulation (PS) at 15 wk (15P) or 21 wk (21P). Blood sampling at 7 h after feeding for all birds and 24 h after feeding for EDS and 48 h after feeding for SAD birds. Values are the means ± SEM with 3 replicate pens of 200 pullets per treatment (n = 6 for PS and RS main effects).

	PS	RS	Wk. 107 h	Wk. 1024 h (EDS) or 48 h (SAD)	Wk. 157 h	Wk. 1524 h (EDS) or 48 h (SAD)	Wk. 2024 h (EDS) or 48 h (SAD)
			pg/mL				
	15P		933 ± 73	1,971 ± 277	862 ± 100	1,532 ± 245	
	21P		1,506 ± 177	3,183 ± 464	1,167 ± 261	2,622 ± 174	1,573 ± 427
		SAD	1,006 ± 200	2,061 ± 278	706 ± 73	1,786 ± 382	
		EDS	1,433 ± 80	3,093 ± 505	1,323 ± 210	2,367 ± 171	
RS × PS	15P	SAD	901 ± 185	1,529 ± 172	691 ± 62	1,051 ± 230	
		EDS	965 ± 58	2,413 ± 399	1,033 ± 132*	2,013 ± 128	
	21P	SAD	1,111 ± 44	2,593 ± 277	721 ± 151	2,522 ± 369	838 ± 164
		EDS	1,901 ± 278*	3,773 ± 692	1,613 ± 346*	2,721 ± 77	2,307 ± 589*

Source of variation			*P* values				
PS			0.01	0.03	0.17	0.00	
RS			0.04	0.06	0.02	0.04	0.00
RS × PS			0.07	0.76	0.21	0.14	

⁎ Value significantly differs from the corresponding RS value for pullets within the same PS curve.

**Table 3:** Coefficient of variation for BW of broiler breeder pullets at various ages raised using different rearing strategies (RS), everyday spin feeding (EDS) or skip-a-day feeding (SAD), to reach target body weight for photostimulation (PS) at 15 wks (15P) or 21 wks (21P). Values are the means ± SEM with 3 replicate pens of 200 pullets per treatment (n = 6 for PS and RS main effects).

	PS	RS	Wk 3	Wk 5	Wk 8		Wk 11	Wk 15	Wk 18		Wk 20
RS × PS					%						
	15P		13.4 ± 0.23	13.8 ± 0.28	12.8 ± 0.28		13.1 ± 0.57	11.0 ± 0.33			
	21P		13.3 ± 0.35	14.1 ± 0.43	14.2 ± 0.44		17.1 ± 0.62	12.4 ± 0.51	12.9 ± 0.49		12.9± 0.41
		SAD	13.1 ± 0.24	13.5 ± 0.24	13.3 ± 0.50		15.9 ± 0.98	12.2 ± 0.63			
		EDS	13.6 ± 0.31	14.4 ± 0.35	13.7 ± 0.53		14.3 ± 1.01	11.3 ± 0.27			
RS × PS	15P	SAD	13.0 ± 0.09	13.4 ± 0.38	12.7 ± 0.33		13.7 ± 0.55	11.2 ± 0.60			
		EDS	13.9 ± 0.62	14.2 ± 0.27	12.9 ± 0.53		12.5 ± 0.96	10.9 ± 0.43			
	21P	SAD	13.2 ± 0.50	13.5 ± 0.39	13.9 ± 0.81		18.0 ± 0.27	13.3 ± 0.75	13.8 ± 0.89		13.9 ± 0.95
		EDS	13.3 ± 0.03	14.4 ± 0.69	14.4 ± 0.71		16.1 ± 0.99*	12.8 ± 0.18	11.9 ± 0.16*		11.9 ± 0.55

Source of variation			*P* values								
PS			0.66	0.59	0.05	0.01		0.02			
RS			0.23	0.19	0.57	0.07		0.09	0.05		0.09
PS × RS			0.37	0.78	0.21	0.70		0.25			

⁎ Value significantly differs from the corresponding RS value for pullets within the same PS curve.

#### EDS vs. SAD Rearing

During rearing the EDS pullets had lower BW than the SAD pullets as they approached the end of the rearing period at 11 and 14 wk of age in the 15P growth curve and wk 18 and 20 in the 21P growth curve (Table 4). EDS feeding has been reported to increase foraging activity in birds (van Middelkoop et al., 2000; de Jong et al., 2005). With EDS feeding, finding feed pellets in the litter is more challenging than with the conventional SAD method and the pullets likely spent more energy foraging for feed in the litter. Zuidhof et al. (2015) also reported a lower overall feed intake associated with EDS feeding. The feed conversion ratio (**FCR**) for both EDS treatments, in their respected growth curves, tended to be higher during rearing. EDS-15 had a numerically higher FCR at the end of rearing, 3.41, compared to SAD-15, 3.30 (*P* = 0.07). EDS-21 also had a higher FCR at the conclusion of rearing, 3.67, compared to SAD-21, 3.55 (*P* = 0.09). Thus, motivating the birds to actively forage in the litter with EDS likely provided an advantage to the more apprehensive birds by increasing both the spatial and temporal availability of feed when compared to SAD, but may also contribute to a higher feed to gain.

**Table 4:** Weekly BW of broiler breeder pullets raised using different rearing strategies (RS), everyday spin feeding (EDS) or skip-a-day feeding (SAD), to reach target body weight for photostimulation (PS) at 15 wks or 21 wks. Values are the means ± SEM with 3 replicate pens of 200 pullets per treatment (n = 6 for PS and RS main effects).

	SAD-15	EDS-15	SAD-21	EDS-21
Wk of age	g/bird			
3	419 ± 4	417 ± 4	419 ± 4	417 ± 4
5	702 ± 7	703 ± 8	624 ± 7	635 ± 6
8	1,105 ± 11	1,047 ± 11	892 ± 8	847 ± 8
11	1,418 ± 12	1,348 ± 8*	1,072 ± 10	1,082 ± 9
14	2,080 ± 9	2,013 ± 8*	1,450 ± 14	1,416 ± 12
18			1,750 ± 16	1,690 ± 9*
20			2,111 ± 12	2,050 ± 8*

⁎ Value significantly differs from the corresponding value for pullets within the same PS curve.

Despite the lower BW, the EDS treatment tended to improve flock BW uniformity at the end of rearing in the 21P growth curve, with EDS-21 having a lower CV than SAD-21 (Table 3). This improved BW uniformity with EDS, likely due to prolonged feeding time, agrees with previous reports (de Jong et al., 2005; Zuidholf et al., 2015). Previous research indicates that enhancement of BW uniformity leads to improved production and makes it easier to meet the nutritional requirements of the flock during rearing and early lay (Hudson et al., 2001). Despite improving BW uniformity, overall, the EDS treatment did not result in lower blood corticosterone levels in the current research. de Jong et al. (2005) also reported that EDS feeding did not improve broiler breeder indices of welfare during rearing

It was hypothesized that the EDS pullets would have lower plasma corticosterone levels than the SAD pullets given they received feed every d and had extended feeding times as they foraged for their food. However, pullets in the SAD treatment had lower corticosterone levels when compared to EDS pullets at both 10 wk of age and 15 wk of age (Table 2). Within the 21P growth curve, SAD-21 pullets had lower plasma corticosterone than EDS-21 pullets 7 h post feeding at both wk 10 and 15. Within the 15P growth curve, SAD-15 pullets had lower blood corticosterone than EDS-15 pullets at 15 wk of age at 7 h post feeding. Plasma corticosterone concentrations increase because of metabolic stress as well as psychological stress, such as frustration (de Jong, et al., 2002). As expected, at both 10 and 15 wk of age, plasma corticosterone was decreased (*P* < 0.05) 7 h post feeding when compared to 24 (EDS) and 48 (SAD) h post feeding. From a metabolic standpoint, it could be argued that the physiology at the end of the fasting period at 24 h for the EDS pullets and at 48 h for the SAD pullets are similar given that the amount of feed fed is doubled for the SAD pullets to sustain them for a fasting period that is also doubled. However, the EDS pullets had higher baseline plasma corticosterone levels at both 7 h after feeding and just prior to feeding than the SAD pullets. Thus, EDS rearing despite providing foraging activity and fed every day is more stressful based on plasma corticosterone values than SAD rearing. This conclusion needs further investigation. It is possible that the metabolic stress is greater in the EDS pullets due to their increased foraging activity which increases their caloric needs. Thus, EDS pullets need to be provided more feed as noted earlier to obtain the same body weight and to equalize the metabolic stress associated with feed restriction. Another potential reason for the increased corticosterone in EDS birds may be attributed to the increased feeding time, compared to SAD birds (observed, but not recorded), which could increase competitive interactions with other birds during prolonged foraging. In addition, as the EDS birds were fed daily by hand scattering of feed on the pen floor, the pullets may have associated human presence in the pen to collect blood samples with feeding even in the case when they had been fed 7 h earlier and were still exhibiting foraging activity. Although human presence in each room to collect blood samples was less than 15 m, the fact that feed was not provided may have led to pullet frustration and increasing levels of stress and corticosterone release.

Once the pullets were moved to the production pens, there were no differences in plasma corticosterone levels through peak egg production based on previous rearing strategies (data not shown).

### Production: Egg Production, Fertility

#### 15P vs. 21P: Egg Production

**Figure 1:**
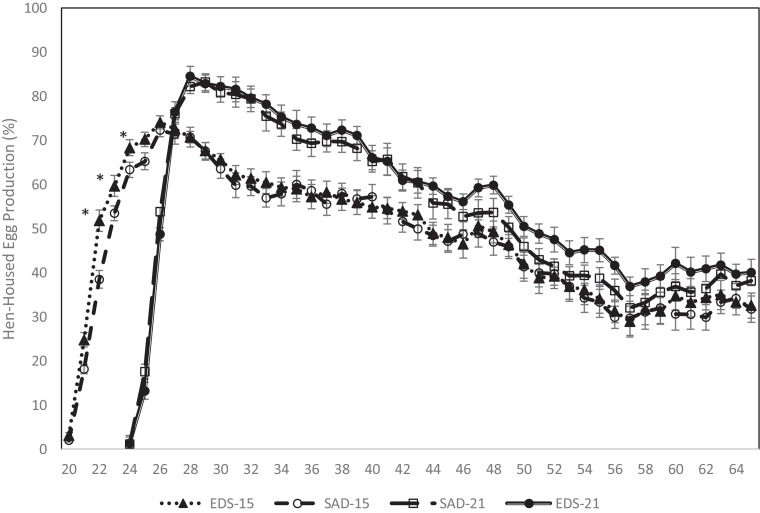
Hen-housed egg production of broiler breeder hens raised using different rearing strategies (RS), everyday spin feeding (EDS) or skip-a-day feeding (SAD), to reach target body weight for photostimulation (PS) at 15 wk or 21 wk. Values are means ± SEM, n = 9 replicate pens of 38 hens for each treatment. Weekly hen-day egg production equals the percentage of hens in lay corrected for mortality. *Means differ (*P* < 0.05) between EDS and SAD in 15P treatment.

**Figure 2:**
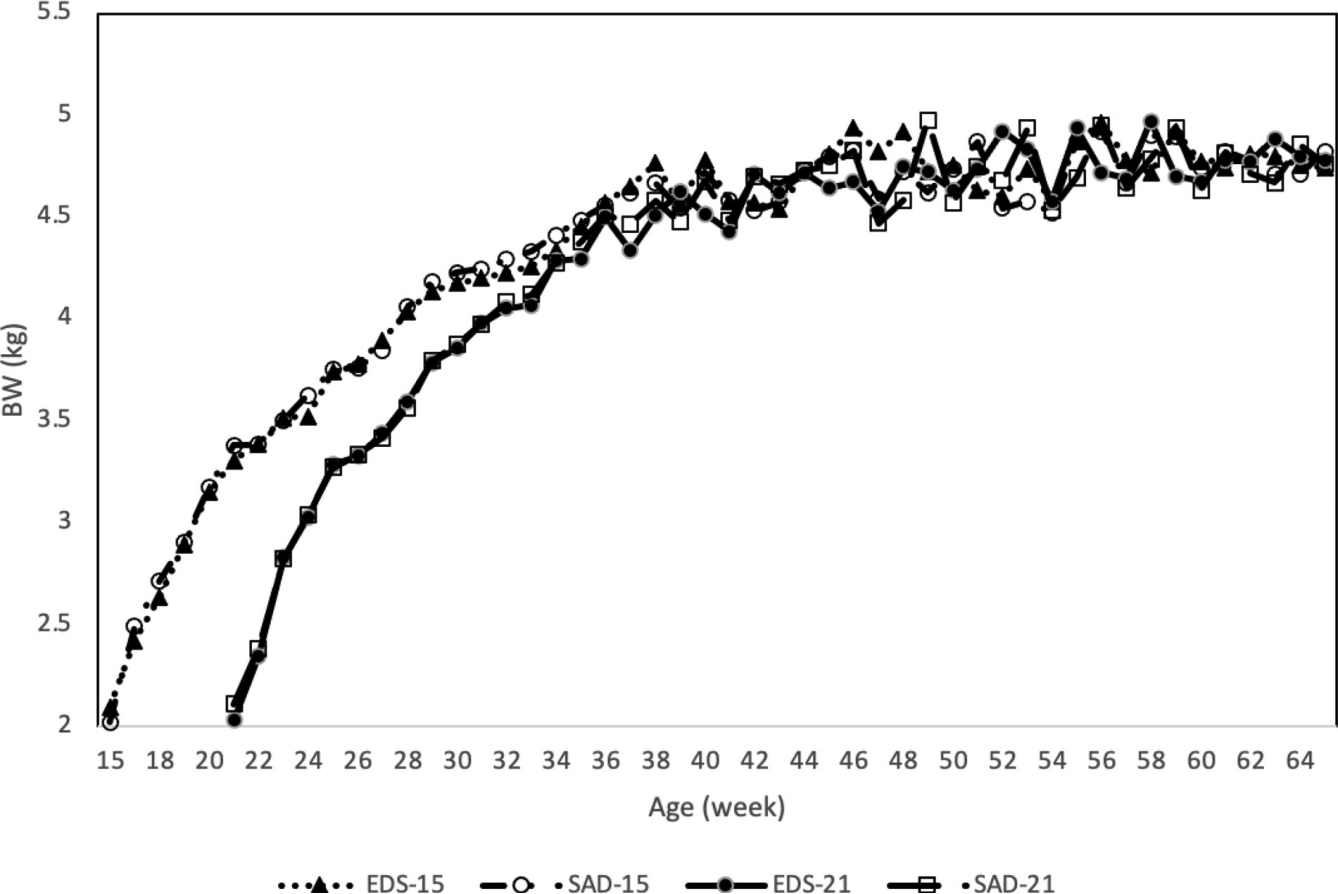
Weekly BW of broiler breeder hens raised using different rearing strategies (RS), everyday spin feeding (EDS) or skip-a-day feeding (SAD), to reach target body weight for photostimulation (PS) at 15 wk (15P) or 21 wk (21P). Values are means ± SEM, n = 3 replicate pens of 38 hens for each feeding treatment. Body weights are calculated on a per-bird basis. Because of the labor involved in weighing all the birds, only one-third of the replicate pens were weighted on a rotating basis for most weeks, but full bird weights (n = 9) were recorded every 10 wk (30, 40, 50, and 60 wk of age).

The advancement of the growth curve (15P) resulted in egg production at an earlier age (20 wk) when compared to the control birds (21P), which commenced egg production at 24 wk of age (Figure 1). This earlier onset of egg production in the 15P birds, as compared to 21P agrees with previous reports on the impact of early PS (Ciacciariello and Gous, 2005a; Renema et al., 2007b) and provides further evidence that faster growth increases the rate of juvenile PR dissipation (Lewis et al., 2007). However, van der Klein et al. (2018), the most recent report on the effect of early PS, reported that breeders brought to target BW for PS at 18 wk of age had an age of first egg at 209 d (29 wk of age) while those with a PS at 21 wk of age had an age of first egg at 180 d (25 wk of age). This discrepancy likely relates to the differences in feeding strategy because van der Klein et al. (2018) used the precision feeding system. In the same van der Klein et al. (2018) research, birds that were fed to a 22% heavier target BW had a similar age of first egg as the 21P hens in the current research. Interestingly, in the current research, the BW at age of first egg for the 15P hens was 3.16 kg at 20 wk of age (Figure 2), which is similar to the age of first egg for the heavier target BW hens photostimulated at 18 wk of age (3.18 kg) in the van der Klein et al. (2018) study. The BW at age of first egg for the 21P hens (3.03 kg) was numerically lighter by 128 g than the BW for 15P hens at age of first egg.

It took the 15P hens 5 wk following PS to lay their first egg, but only 3 wk for the 21P hens to lay their first egg. This increased time interval between PS and age of first egg associated with early PS agrees with previous reports (Robinson et al., 1996; Ciacciariello and Gous, 2005b; Renema et al., 2007a; Pishnamazi et al., 2014; van der Klein et al., 2018). Peak egg production occurred 11 and 8 wk after PS for the 15P and 21P hens, respectively (Figure 1). Peak HHEP was greater (*P* < 0.00) in the 21P hens (83 ± 1.0 %) at 29 wk of age than the 15P hens (73 ± 1.5 % HHEP) at 26 wk of age. Although peak egg production was lower for the 15P hens, their early onset of lay resulted in them producing an equal amount of total eggs/hen as the 21P hens (Table 5). This lower peak production for 15P follows previous reports showing a reduced peak, and lower overall egg production, associated with early PS (Ciacciariello and Gous, 2005b; van der Klein et al., 2018).

**Table 5:** Average total eggs per hen and classification of all eggs produced through 60 wk of age by broiler breeder pullets raised using different rearing strategies (RS), everyday spin feeding (EDS) or skip-a-day feeding (SAD), to reach target body weight for photostimulation (PS) at either 15 wks (15P) or 21 wks (21P). Values are the means ± SEM with 9 replicate pens of 38 hen per treatment (n = 18 for PS and RS main effects).

			Total	Settable	Cracked	Floor	Double-yolked	Dirty	Misshaped
RS × PS	PS	RS	#/hen	%					
	15P		151 ± 6.9	83.2 ± 0.75	4.3 ± 0.24	2.7 ± 0.33	1.1 ± 0.06	8.2 ± 0.7	1.6 ± 0.10
	21P		160 ± 7.2	84.2 ± 0.53	3.7 ± 0.19	3.8 ± 0.35	0.5 ± 0.09	7.2 ± 0.33	1.3 ± 0.09
		SAD	152 ± 11.2	84.2 ± 0.64	3.9 ± 0.21	2.8 ± 0.37	0.8 ± 0.13	7.5 ± 0.43	1.5 ± 0.09
		EDS	159 ± 9.8	83.1 ± 0.68	4.0 ± 0.27	3.8 ± 0.31	0.9 ± 0.12	6.9 ± 0.19	1.4 ± 0.11
RS × PS	15P	SAD	149 ± 5.9	83.8 ± 0.96	4.0 ± 0.21	2.3 ± 0.56	1.1 ± 0.05	8.0 ± 0.50	1.7 ± 0.13
		EDS	153 ± 6.4	82.5 ± 0.98	4.5 ± 0.27	3.1 ± 0.31	1.1 ± 0.07	8.3 ± 0.88	1.4 ± 0.13
	21P	SAD	156 ± 5.6	84.7 ± 0.83	3.8 ± 0.20	3.2 ± 0.46	0.5 ± 0.10	6.9 ± 0.19	1.5 ± 0.18
		EDS	164 ± 5.9	83.7 ± 0.66	3.6 ± 0.15	4.5 ± 0.46	0.6 ± 0.10	7.5 ± 0.42	1.2 ± 0.13

Source of variation			*P* values						
PS			0.09	0.29	0.07	0.02	0.00	0.09	0.05
RS			0.39	0.24	0.61	0.04	0.78	0.46	0.31
PS × RS			0.67	0.91	0.36	0.61	0.51	0.84	0.85

*Value significantly differs from the corresponding RS value for hens within the same PS curve.

The average number of settable eggs per hen housed did not differ among any of the treatments (Table 5). Ciacciariello and Gous (2005b) previously reported a significant decrease in settable eggs with PS at 15P but didn't distinguish or classify the unsettable eggs. In the current research, the number of floor eggs was greater in the 21P treatment than the 15P treatment (Table 5). The consistent decrease in floor eggs throughout the production cycle associated with early PS, to our knowledge, has not been seen in other studies concerning early PS and egg production. The number of double yolk (**DY**) eggs produced was greater in the 15P hens than the 21P birds (Table 5). This difference in DY egg production between the 15P and 21P hens occurred in the period from the onset of egg production until peak egg production.

A positive relationship between DY egg production associated with earlier sexual maturation and heavier body weights is well established in broiler breeder hens (Lewis et al., 1997; Heck et al., 2004; Ciacciariello and Gous, 2005b; Hocking and Robertson, 2005). Even with only moderate overfeeding, the number of hierarchical follicles at sexual maturity can increase and predisposition the birds to increased DY egg production (Hocking et al., 1987; Robinson et al., 1998). Renema et al. (2007a) also found an increase in hierarchical follicles associated with early PS at 18 wk of age. The results from the current research suggest that although BW at the onset of egg laying may contribute to the increase in DY eggs other factors are likely involved. The 15P hens weighed 3.161 kg at the onset of lay at 20 wk of age and 3.769 kg when peak egg production occurred at 26 wk of age, and thus BW increased by 608 g during this 6 wk period. In contrast, the 21 P hens weighed 3.033 kg at the onset of lay at 24 wk of age and 3.790 kg when peak egg production occurred at 29 wk of age and thus BW increased by 757 g in this 5-wk period. Therefore, while the 15P hens weighed 128 g more than the 21P hens at the onset of lay, which may have contributed to them laying more DY eggs, the fact that their subsequent rate of BW gain to peak egg production is less than that of the 21P hens make it unlikely that BW was the only contributing factor in the increase in DY egg production.

In addition to DY egg production differences between the 15P and 21 P hens through peak egg production, egg weigh differences between these 2 treatments during this period also indicate that early PS may impact early follicular development. Although overall average egg weight did not differ across the entire production period between the 15P and 21P hens (data not shown), the 15P hens produced lighter eggs during their first 5 wk of production. Three weeks after the onset of egg production (23 and 27 wk of age for the 15P and 21P hens, respectively), mean egg weight was 48.8 g and 50.5 g (*P* < 0.001) for the 15P and 21P hens, respectively with corresponding hen BW at 3.509 and 3.428 kg. At peak egg production (26 and 29 wk of age for the 15P and 21P hens, respectively), mean egg weight was 53.5 g and 54.0 g (*P* < 0.090) for the 15P and 21P hens, respectively with the corresponding hen BW at 3.769 and 3.790 kg. Finally, at 31 wk of age mean egg weight was 58.3 g and 58.2. g (*P* = 0.852) for the 15P and 21P hens, respectively with the corresponding hen BW at 4.202 and 3.836 kg. In the current research, BW does not explain the differences in egg weight, even though egg weight is thought to be positively correlated with hen weight or hen weight at sexual maturity (McDaniel et al., 1981), and given Pishnamazi et al. (2014) concluded that egg weight differences between hens with PS at different ages were attributed to the difference in BW at sexual maturity, not by the effect of PS age. The rate of lay could also impact egg weight since hens that lay less may have a higher rate of yolk deposition per follicle and thus higher overall egg weights (McLeod et al., 2014; Tumova et al., 2017), but peak egg production was lower in the 15P hens compared to the 21P hens.

The current research indicates that early PS leads to decreased egg weights in early production. Studies by Ciacciariello and Gous (2005b) and Renema et al. (2007a) also found that initial egg production with earlier PS is associated with the production of smaller eggs. Although the current study did not record ovarian measurements, Renema et al. (2007a) found a reduction in F1 weight associated with early PS at 18 wk of age. A simultaneous increase in the production of DY eggs and a decrease in average egg weight found in the current research with early PS agreed with the report by Ciacciariello and Gous (2005b).

#### EDS vs. SAD: Egg Production

Overall, there were not differences in total egg production per hen in relation to the rearing feed strategy between EDS and SAD (Table 5). However, hens that had been reared using the EDS feeding strategy produced more floor eggs than the SAD-reared hens (Table 5). It is possible that the litter foraging activity associated with EDS rearing causes a predilection for these hens to want to lay eggs in the litter. Regretfully, in the current research floor eggs were not further categorized as floor eggs produced on the slats or in the litter.

#### Fertility and Hatch

There were no significant differences in overall fertility and hatchability between PS and rearing treatments (Table 6). The results demonstrate that both roosters and hens can dissipate juvenile PR to allow successful PS at 15 wk of age and that early PS does not impact flock fertility. This result agrees with Robinson et al. (1996), who also reported no differences in fertility and hatchablity associated with early PS. More recently, Shi et al. (2021) also reported no differences in fertility, hatchability, semen quality and sperm count associated with roosters that were PS as early as 16 wk of age.

**Table 6:** Overall fertility and hatchability of broiler breeder eggs incubated from hens with different rearing strategies (RS), everyday spin feeding (EDS) or skip-a-day feeding (SAD), to reach target body weight for photostimulation (PS) at either 15 wk (15P) or 21 wk (21P)^1^.

	PS	RS	Fertility	Hatchability^2^	Hatch of fertile	Early dead^3^^,^^4^	Late dead^3^^,^^4^
RS × PS			%				
	15P		84 ± 2.4	80 ± 2.5	95 ± 1.0	1.4 ± 0.46	1.6 ± 0.51
	21P		84 ± 2.7	80 ± 2.7	93 ± 1.2	1.3 ± 0.43	1.9 ± 0.56
		SAD	84 ± 2.4	79 ± 2.4	94 ± 1.0	1.2 ± 0.19	1.7 ± 0.49
		EDS	84 ± 2.5	80 ± 2.4	94 ± 1.1	1.5 ± 0.24	1.7 ±0.51
RS × PS	15P	SAD	84 ± 2.4	80 ± 2.5	95 ± 1.0	1.4 ± 0.41	1.7 ± 0.48
		EDS	83 ± 2.5	79 ± .2.7	94 ± 1.0	1.5 ± 0.44	1.6 ± 0.56
	21P	SAD	83 ± 2.4	79 ± 2.4	93 ± 1.1	1.1 ± 0.43	1.8 ± 0.52
		EDS	86 ± 2.3	82 ± 2.6	93 ± 1.0	1.5 ± 0.49	1.9 ± 0.51

Source of variation			*P* values				
PS			0.61	0.76	0.21	0.69	0.26
RS			0.65	0.68	0.98	0.42	0.98
PS × RS			0.36	0.37	0.78	0.61	0.70

1 Values are means ± SEM; n = 15 replicate pens of 35 hens each for both feeding treatments. Ninety eggs from each replicate pen were incubated and hatched every 3 wk.

2 Hatch of eggs set.

3 Embryo mortality was classified as early dead (0–12 d) or late-dead (13–21 d of incubation).

4 Calculated as a percentage of fertile eggs.

#### Mortality

The cumulative number of hens that had died at 25, 45, and 65 wk of age for the 15P SAD treatment was 8, 34, and 75 respectively, for the 15P EDS treatment it was 12, 34, and 89, respectively, for the 21P SAD treatment it was 3, 32, and 66, respectively and for the 21P EDS treatment it was 1, 22, and 41. Mortality was primarily associated with rooster injury, infection from internal ovulations and leg/mobility issues, and thus given the 6 extra wk of production it is not surprising that the 15P treatment had a greater number of dead hens than the 21P treatment by the end of the experiment.

## Conclusions

Advancing the onset of PS and EDS feeding were investigated as mechanisms to reduce rearing stress in broiler breeder hens in the current research. As indicated by plasma corticosterone levels, EDS increases stress levels relative to traditional SAD feeding. This may be metabolically related in part, as the activity of foraging appears to increase energy demands as seen by the lower BW of these pullets at the end of rearing. Thus, if EDS is to be further evaluated providing them more feed than SAD pullets would be recommended. The current research indicated that PR can be dissipated to an extent that permits PS at 15 wk of age and this advancement allows for less severe feed restriction to meet target BW for reproduction 6 wk earlier. Feeding on a less restrictive growth curve for early PS not only improved body weight uniformity, but it significantly decreased corticosterone levels during rearing and did not affect subsequent fertility and hatch of fertile eggs. Although total egg production was comparable due to the extra 4 wk of egg production some of the results indicate that some influence of PR may still exist following PS at 15 wk. Further research is needed to see if subsequent refinement of this early PS strategy could overcome the lower peak egg production, and the decrease in egg weight and increase in DY egg production proceeding peak egg production observed in the current research.
